# Dissecting the roles of thymoquinone on the prevention and the treatment of hepatocellular carcinoma: an overview on the current state of knowledge

**DOI:** 10.1186/s13027-019-0226-9

**Published:** 2019-04-16

**Authors:** Sabrina Bimonte, Vittorio Albino, Antonio Barbieri, Maria Luisa Tamma, Aurelio Nasto, Raffaele Palaia, Carlo Molino, Paolo Bianco, Andrea Vitale, Rita Schiano, Aldo Giudice, Marco Cascella

**Affiliations:** 1 Division of Anesthesia and Pain Medicine, Istituto Nazionale Tumori, IRCCS - Fondazione G Pascale, Naples, Italy, Naples, Italy; 2 Division of Abdominal Surgical Oncology, Hepatobiliary Unit, Istituto Nazionale Tumori, IRCCS - Fondazione G Pascale, Naples, Italy, Naples, Italy; 3 S.S.D. Sperimentazione Animale, Istituto Nazionale Tumori, IRCCS Fondazione G. Pascale, Naples, Italy; 4 U. O. C. di Chirurgia Generale ad indirizzo Oncologico P.O. “A. Tortora”, Pagani, Salerno, Italy; 5 Chirurgia Generale AORN A, Cardarelli, Naples, Italy; 6 Pineta Grande Hospital, Caserta, Italy; 7 Epidemiology Unit, IRCCS Istituto Nazionale Tumori “Fondazione G. Pascale”, 80131 Naples, Italy

**Keywords:** *Nigella sativa*, Thymoquinone, Hepatocellular carcinoma, Cell proliferation, Cell apoptosis

## Abstract

Thymoquinone (TQ) is the principal active monomer isolated from the seed of the medicinal plant *Nigella sativa*. This compound has antitumor effects against various types of cancer including hepatocellular carcinoma (HCC), mainly due to its anti-inflammatory and anti-oxidant properties. Several pre-clinical studies showed that TQ, through the modulation of different molecular pathways, is able to induce anti-apoptotic and anti-proliferative effects in HCC, without signs of toxicity. Moreover, it has been suggested that TQ has hepatoprotective effects by enhancing the tolerability and effectivity of neoadjuvant therapy prior to liver surgery, although the underlying mechanisms are not completely understood. Based on these findings, is assumable that TQ could represent a valuable therapeutic option for patients suffering from HCC. In this review, we summarize the potential roles of TQ in the prevention and treatment of HCC, by revising the preclinical studies and by highlighting the potential applications of TQ as a therapeutic choice for HCC treatment into clinical practices.

## Background

Thymoquinone (TQ) is the predominant bioactive constituent present in the volatile oil of black seed (*Nigella sativa*), particularly used as a condiment in the Middle East [1–4]. Accumulating of evidence showed that TQ has anti-oxidant effects and anti-proliferative effects in many types of cancer, including liver tumors, without signs of toxicity to normal cells [5–11]. Moreover, it has been proved that TQ and *Nigella sativa* possess hepatoprotective effects by enhancing the tolerability and the effectivity of neoadjuvant therapy prior to liver surgery [12–16]. As regards to hepatocellular carcinoma (HCC), due to the unavailability of successful therapy for HCC patients mainly for those at an advanced stage of disease [17–20], new alternative therapies based on the use of natural compounds as a supplement to conventional schedules for HCC treatment, should be taken into account [21]. Based on these findings, is assumable that TQ could be considered a therapeutic option for the prevention and the treatment of HCC. For this purpose, we summarize the potential roles of TQ in the prevention and treatment of HCC, by revising the preclinical studies and by highlighting the potential applications of TQ as a therapeutic choice for HCC treatment into clinical practices.

### TQ: chemical structure, biological properties, and roles in the human hepatocellular carcinoma

**Fig. 1:**
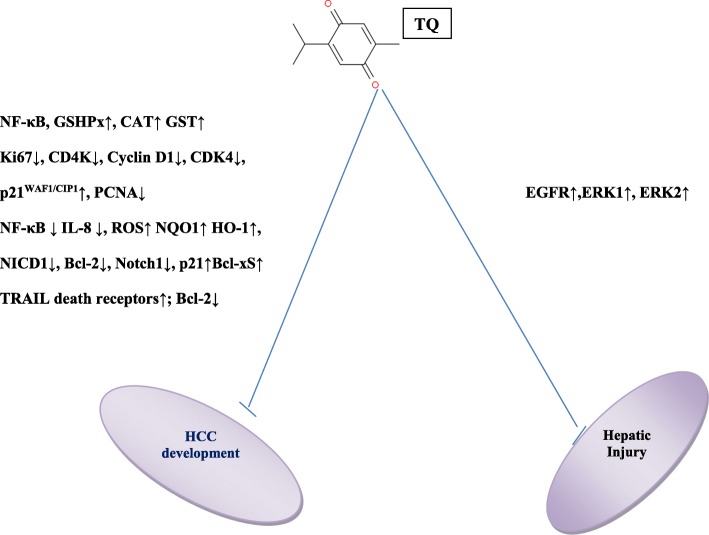
Key signaling pathways that regulate the function of TQ in HCC develepoment and in the hepatic injury. The cartoon recapitulates the principle signaling pathways by which TQ blocks hepatocellular carcinoma growth (**a**) and the hepatic injury (**b**). Abbreviations: GSHPx: glutathione peroxidase; CAT: catalase; GST: glutathione-s-transferase; Ki67: cell proliferating markers(Labeling index); PCNA: proliferating cellular antigen; CDK4: Cyclin-Dependent Kinase 4; NF-κB: Nuclear factor kappa-light-chain-enhancer of activated B cells; ROS: reactive oxygen species; NQO1: NADFH quinone oxidoreductase 1; HO-1: Heme oxygenase isoenzime-1; TRAIL: Tumor necrosis factor-related apoptosis-inducing ligand; Bcl-2: BCL2, Apoptosis Regulator; Notch1: Neurogenic locus notch homolog protein 1; NICD1: notch intracellular domain 1; BAX: Bcl-2 Associated X, Apoptosis Regulator

Thymoquinone (TQ) or 2-isopropyl-5-methyl-1, 4-benzoquinone (Fig. 1), is the predominant constituent derived from the seeds of *Nigella sativa* whose composition has been previously described by Mollazadeh et al [14]. Many in vivo and in vitro reports have demonstrated the therapeutic efficacy of TQ against a wide range of cancer including ovarian cancer, breast cancer [22], pancreatic cancer [23], lung cancer [24], fibrosarcoma [25], neuroblastoma [26], osteosarcoma [27] and myeloma [28]. Due to its chemical structure, TQ is able to act as a free radical and as a superoxide radical scavenger [29]. TQ exerts its anti-inflammatory and immunomodulatory effects by acting on specific signaling pathways as nuclear factor kappa-light-chain-enhancer of activated B cells (NF-kB), interleukin 1- beta (IL-1β), TNF-α (Tumor necrosis factor-alfa) [30]. Regarding HCC, it has been reported that TQ is able to inhibit tumor growth by regulating the cell cycle progression and the apoptosis, through the repression of the Notch signaling pathway [31]. In addition, TQ and *Nigella sativa* have a chemopreventive role in HCC by inhibiting the EGFR/ERK1/2 signaling pathway (Fig. 1) [15].

### Pre-clinical shreds of evidence on the role of TQ in HCC cell growth: a current state of the art

Accumulating pieces of pre-clinical evidence shed a light on the inhibitory role of TQ in HCC initiation and progression. The first study has been conducted on rats with hepatocarcinogenesis-induced by diethylnitrosamine (DENA, 200 mg/kg, I.P.) [32]. The authors showed that TQ (4 mg/kg/day delivered in drinking water) was able to counteract the DENA-induced initiation of liver cancer. Particularly, TQ restored the biochemical values and the histopathological damages in liver tissues induced by DENA. Moreover, due to its anti-oxidant properties, TQ preserved the activity and mRNA expression of antioxidant enzymes as nitrate/nitrite (NOx), glutathione (GSH), glutathione peroxidase (GSHPx), glutathione-s-transferase (GST) and catalase (CAT) which were altered by DENA (Table 1). Subsequently, in a fascinating study, et al. [33], showed that TQ (20 mg/kg) had anti-proliferative effects in experimental rats with hepatocarcinogenesis induced by N-nistrodiethhylamine (NDEA, 0,01% in drinking water), by arresting the cycle progression at G1/S phase. Specifically, TQ reduced the levels of liver injury and tumor markers and restored the normal parenchymal liver architecture which was altered by NDEA-administration. Finally, the anti-proliferative effect of TQ was assessed firstly, by evaluating the expression profile of cell proliferating markers, Ki67 and proliferating cellular antigen (PCNA), secondly, by analyzing the TQ’s action on the regulation of cell cycle progression on G1/S phase. This latter aim was achieved by evaluating the expression of typical cell cycle proteins, CD4K, Cyclin D1, CDK4, and p21^WAF1/CIP1^, by the experiment of immunoprecipitation on rats of each group of treatment (Table 1). Results from these studies suggested that TQ was able to suppress hepatocellular tumor growth by inhibiting the proliferation and by arresting the cell cycle progression on the G1/S phase.

**Table 1:** A summary of pre-clinical studies on the role of TQ in hepatocellular carcinoma cell growth

Cell lines	Animal model	Dose of TQ	Molecular targets	Reference
	Rats with Hepatocarcinogenesis-induced by diethylnitrosamine (DENA 200 mg/kg, I.P.).	50 mg/L in drinking water = 4 mg/kg daily for 7 consecutive days.	GSHPx↑, CAT↑ GST↑	[32]
	Rats with hepatocarcinogenesis induced by N-nistrodiethhylamine (NDEA, 0,01% in drinking water)	20 mg/kg body weight daily from the 3th to 5th week of treatments. (oral gavage)	Ki67↓, PCNA, Cyclin D1↓, CDK4↓ and p21^WAF1/CIP1^↑,	[33]
HepG2		12.5, 25 or 50 M μM for 24 h.	NF-κB↓, IL-8 ↓, ROS↑ NQO1↑ HO-1↑, Bcl-xS↑, TRAIL death receptors↑; Bcl-2↓	[34]
Hep3B, SMMC7721, HepG2, Bel7402, MHCC97-L, MHCC97-H, HHCC		20,40,60, 80 μM from 24 to 72 h.	Bcl-2↓, Notch1↓, NICD1↓, Jagged1↓, Hes1↓ cyclin D1↓, CDK2↓ p21↑, Bax↑	[31]
	Liver tumor xenografts in athymic nude mice (Hep cells)	5 mg/kg daily (subcutaneously injected); 20 mg/kg daily (subcutaneously injected) for 31 days.	NICD1↓, Bcl-2↓, Notch1↓, p21↑	[31]

*Abbreviations*: *GSHPx* glutathione peroxidase, *CAT* catalase, *GST* glutathione-s-transferase, *Ki67* cell proliferating markers(Labeling index), *PCNA* proliferating cellular antigen, *CDK4* Cyclin-Dependent Kinase 4, *NF-κB* nuclear factor kappa-light-chain-enhancer of activated B cells, *ROS* reactive oxygen species, *NQO1* NADFH quinone oxidoreductase 1, *HO-1* Heme oxygenase isoenzime-1, *TRAIL* tumor necrosis factor-related apoptosis-inducing ligand, *Bcl-2 BCL2, Apoptosis Regulator*, *Notch1* neurogenic locus notch homolog protein 1, *NICD1* notch intracellular domain 1, *Jagged1* protein jagged-1, *Hes1* Hes Family BHLH Transcription Factor, *CDK2* Cycle

Similar findings were reported by Ashour et al in vitro experiments on HepG2 cells [34]. Specifically, TQ was able to inhibit the growth of HCC cells by arresting cell cycle on G2M phase and by activating the expression of caspase-3 and caspase-9 and the cleavage of poly (ADP-ribose) polymerase. Moreover, TQ was able to enhance the TRAIL-induced death of HepG2 cells, thought the up-regulation of death receptors, the inhibition of Nuclear factor kappa-B (NF-κB) and interleukin-8 (IL-8), the stimulation of reactive oxygen species (ROS) and mRNAs of NAD(P)H quinone dehydrogenase 1 (NQO1) and heme oxygenase 1 (HO-1). These results suggest that TQ could be considered a potential substance for the prevention and the treatment of HCC. In a fascinating in vitro and in vivo studies, a pivotal role of TQ in the inhibition of HCC cell growth was also reported [31]. Basically, the authors showed a retarded tumor cell growth induced by TQ treatment accompanied by arresting the cell cycle in G1 phase (SMMC7721 cells) or in S phase (Hep3B cells) and by upregulating p21 and downregulating CDK2 and cyclinD1 expression according to TQ concentrations. Moreover, TQ enhanced apoptosis by decreasing Bcl-2 expression and increasing Bax expression. These findings were confirmed in a xenograft mouse model of HCC. Particularly, tumors of xenograft liver mice showed a decreased expression of NICD1 and Bcl-2 levels while an increment of p21 expression was observed. Altogether, these data suggest that TQ inhibits HCC growth by inhibiting the Notch signaling pathway.

### Mechanism of action: a link between the hepatoprotective effects of TQ and its antioxidant properties

Despite the inhibitory role of TQ on HCC cell growth, several in vivo reports on different liver models [13, 35–53], shed a light on the hepatoprotective effects of TQ, commonly associated to its antioxidant properties. In a fascinating systematic review, Tekbas et al. [13] suggested that TQ, due to its multiple properties, could be considered as a new substance that reduced the hepatic injury.

It is well assumed, that liver injury is commonly associated with changes in the expression of the principal liver enzymes and in liver tissue damage which is commonly attributed to an oxidative stress [13]. Results from the above-mentioned studies on different liver models suggest that TQ has a hepatoprotective role by increasing the resistance to oxidative stress, through the regulation of the oxidative markers content.

Specifically, TQ is able to prevent malondialdehyde (MDA) production [37–45], to block the lipid peroxidation [38, 46–49], to reduce the content of nitric oxide (NO) [50, 52] and to decrease the concentration of GSH [39, 43, 44, 49–51, 53]. The underlying mechanism is mainly based on the inhibition of oxygen free radicals production induced by TQ, which in turn regulates the inflammatory molecular pathways as NF-kB, tumor necrosis factor (TNF-α), interleukin (IL-1β) and the nitric oxide signaling pathway.

An interesting study on the protective mechanism of TQ on HCC was recently demonstrated in rats with HCC induced by diethylnitrosamine (DENA) [32]. The authors identified the EGFR/ERK1/2 signaling pathway as the underlying mechanism by which TQ exerted the hepatoprotective function. Moreover, TQ was able to protect liver thanks to its antioxidant properties by enhancing the activity of superoxide dismutase (SOD), glutathione peroxidase (GPx), catalase (CAT) and glutathione- s-transferase (GST).

Taken together, these findings, suggest that TQ could be considered not only a potential drug for the prevention and the treatment of HCC but also as a hepatoprotective agent in HCC patients.

## Conclusions

Several pre-clinical studies depicted here demonstrated that TQ induces apoptosis and restrains HCC progression by acting on different molecular pathways. These findings largely support the use of TQ into clinical practice for HCC counteraction and treatment. Despite TQ compound is currently used in clinical trials for the treatment of different type of cancer and other diseases, no clinical trials have been performed, until now, for patients suffering from HCC. For these reasons, more studies are extremely needed. These examinations ought to be engaged 1) on the understanding of the molecular mechanism regulated by TQ in HCC; 2) on the identification of the optimum therapeutic dosage of TQ for intervention trials in HCC patients.

## Abbreviations

ADP: Poly-ribose polymerase
BAX: Bcl-2 Associated X, Apoptosis Regulator
Bcl-2: BCL2, Apoptosis Regulator
Bcl-xS: B-cell lymphoma-extra small
BHLH: Transcription Factor
CAT: Catalase
CDK2: Cyclin-Dependent Kinase 2
CDK4: Cyclin-Dependent Kinase 4
DENA: Diethylnitrosamine
EGFR: Epidermal growth factor receptor
ERK-1: Extracellular signal-regulated kinase 1
ERK-2: Extracellular signal-regulated kinase 2
GPx: Glutathione peroxidase
GSH: Glutathione
GST: Glutathione-s-transferase
HCC: Hepatocellular carcinoma
Hes1: Hes Family
Hes1: Hes Family BHLH Transcription Factor
HO-1: Heme oxygenase isoenzime-1
IL-1β: Interleukin 1- beta
IL-8: Interleukin-8
Jagged1: Protein jagged-1
Ki67: Cell proliferating markers(Labelling index)
MDA: Malondialdehyde
NDEA: N-nistrodiethhylamine
NF-κB: Nuclear factor kappa-light-chain-enhancer of activated B cells
NICD1: Notch intracellular domain 1
NICD1: the released notch intracellular domain 1
Notch1: Neurogenic locus notch homolog protein 1
NOx: Nitrous Oxides
NQO1: NADFH quinone oxidoreductase 1
PCNA: Proliferating cellular antigen
ROS: Reactive oxygen species
SOD: Superoxide-dismutase
TNF-α: Tumour necrosis factor-alfa
TQ: Thymoquinone
TRAIL: Tumour necrosis factor-related apoptosis-inducing ligand
